# Status and influencing factors of health literacy among college students of traditional Chinese medicine: a cross-sectional study

**DOI:** 10.3389/fpubh.2025.1542764

**Published:** 2025-02-10

**Authors:** Jie Zhu, Cuixia Lin, Yanhui Yang, Rui Yao, Ran Yang, Mingqian Jiang

**Affiliations:** ^1^School of Nursing, Shandong University of Traditional Chinese Medicine, Jinan, Shandong, China; ^2^School of Health, Shandong University of Traditional Chinese Medicine, Jinan, Shandong, China

**Keywords:** university of traditional Chinese medicine, college students, health literacy, influencing factor, healthy school

## Abstract

**Background:**

College students, as the future strength of national development, have a high level of health literacy in line with the goal of “Healthy China”, but the current research found that the health literacy level of college students is uneven and needs to be improved, and there is still a lack of research on the health literacy of students in colleges and universities of traditional Chinese medicine (TCM). Therefore, this study aims to assess the health literacy level of students in TCM colleges and analyze its influencing factors, so as to provide a basis for implementing effective health literacy intervention measures and building healthy schools.

**Methods:**

Using the convenient sampling method, 925 college students in a TCM university in Shandong Province were selected to conduct an online electronic questionnaire survey using “Questionnaire Star” from December 2022 to March 2023. SPSS 22.0 software was used to process and analyze the data. Descriptive statistical analysis was used to analyze the general data of the respondents, chi-square test was used to compare the count data between groups, and Logistic regression model was used to analyze the influencing factors of health literacy.

**Results:**

The health literacy level of TCM college students was 57.30%. Among the three aspects of health literacy, the level from high to low was: healthy lifestyle and behavior literacy (65.41%), health skill literacy (58.70%) and basic knowledge and concept literacy (53.62%). Among the six categories of health literacy, the level of scientific health literacy (79.68%) was the highest, followed by safety and first aid literacy (72.86%), health information literacy (61.62%), basic medical literacy (61.30%), chronic disease prevention literacy (60.11%) and infectious disease prevention literacy (40.86%). The results of multifactorial logistic regression analyses showed that females (AOR: 1.92; 95%CI: 1.40–2.62), college seniors (AOR: 2.02; 95%CI: 1.01–4.05), never smokers (AOR: 2.99; 95%CI: 1.57–5.72), and awareness of the concept of “health literacy” (AOR: 1.54; 95%CI: 1.11–2.13) were protective factors for health literacy, and the health literacy level of students in TCM colleges and universities was statistically significantly positively correlated with their health literacy level (*p* < 0.050).

**Conclusion:**

Compared with most studies at home and abroad, the overall level of health literacy among students of TCM colleges is high. The healthy lifestyle and behavior literacy of students in TCM colleges is better than knowledge and skill literacy, which is manifested as the separation of knowledge and practice. Gender, grade, smoking or not, being aware of the concept of “health literacy” are the influencing factors of health literacy level of students in TCM colleges.

## 1 Introduction

Health literacy refers to the ability of individuals to access and understand basic health information and services, and to use this information and services to make good decisions to maintain and promote their health (1). Domestic and foreign scholars intervene in people’s health literacy based on the theories of “knowledge, attitude and practice model” (a set of behavioral intervention models through the acquisition of knowledge, attitude change and behavior formation) and “health belief model” (this model believes that when individuals have sufficient health beliefs, they will take corresponding health behaviors) (2, 3). In order to further improve people’s health literacy, research on the theoretical framework and assessment tools of health literacy has gradually increased in recent years, especially the research on health literacy of special populations has received increasing attention (4, 5). The 14th Five-Year National Health Plan points out that health problems and influencing factors should be comprehensively intervened to further improve the health literacy level of residents (6). As the main force of national and social development, college students’ health literacy level has an important impact on the health literacy level of residents. After the COVID-19 pandemic, the government has paid more attention to improving the health literacy level of students. Therefore, the Ministry of Education proposed the implementation of the National Healthy Schools Construction Plan (7).

Previous studies have reported the problems related to health literacy of college students (8, 9), and there is a large gap in the health literacy level of college students. Low health literacy means low awareness and ability to seek health information. Such college students often have health problems such as smoking, low self-esteem, loneliness and so on (10, 11). There are also rich related studies on health literacy of college students in western medicine colleges (12, 13), but there are few studies on the health literacy of college students in traditional Chinese medicine (TCM), which limits the colleges of TCM to develop accurate plans or implement health promotion activities to improve the health literacy level of students. In 2021, a TCM college conducted an eHealth literacy survey on 1,007 undergraduates, and the results showed that the level of eHealth literacy of college students of TCM was low (14). Compared with western medicine colleges and universities, TCM colleges and universities have unique advantages, they pay more attention to health care services and advocate “preventive treatment of disease” (15), which is in line with the concept of “Healthy China” (6). Therefore, it is necessary to carry out the research on the status of health literacy of students in TCM colleges and universities and explore the influencing factors of health literacy. This study conducted a sampling survey of college students in a TCM university in Shandong Province, in order to explore the health literacy level and related influencing factors of college students in TCM, so as to provide reference for carrying out targeted health literacy promotion and healthy school construction in the future.

## 2 Materials and methods

### 2.1 Study area and period

This study was conducted from December 2022 to March 2023 in a TCM university in Shandong Province, China.

### 2.2 Study design and participants

The convenience sampling method was used to select undergraduates in a TCM university in Shandong Province as the research object for a cross-sectional study. Inclusion criteria: ① Undergraduate; ② Age ≥ 18 years old. Exclusion criteria: ① those who did not complete the questionnaire or withdrew during the course. ② too long or too short time to answer the questionnaire (according to ^−^x ± s, < 219.62 s or > 1062.51 s); ③ There were obvious regularity or abnormal values in the content of the questionnaire. The purpose and significance of the study were explained to the subjects before the study, and the investigation was conducted after obtaining the consent of the subjects.

### 2.3 Measures

#### 2.3.1 Questionnaire survey

The electronic questionnaire was used to conduct the survey. The related questions of the self-designed questionnaire came from the “Health literacy of Chinese citizens - Basic knowledge and Skills (2015 edition)” (16) and the “National Residents’ Health literacy Questionnaire” prepared by the China Health Education Center (17). The questionnaire included two parts: general information of the respondents and questions related to health literacy. The present study was modified appropriately in terms of demographics. Health literacy related issues can be divided into three aspects: basic knowledge and concept, healthy lifestyle and behavior, and health skills. According to the public health problems, health literacy can be divided into six types of health problems: scientific health concept, infectious disease prevention, chronic disease prevention, safety and first aid, basic medical care and health information (17). There were 51 questions in this survey. The judgment questions and single-choice questions were scored 1 point per question, the multiple-choice questions were scored 2 points per question, and the wrong answers or underanswers were scored 0 points, with a total score of 65 points. According to the criterion of health literacy, if the score of the questionnaire reaches 80% or more of the total score (≥48 points), it can be considered to have health literacy, and the criterion of literacy level of three aspects and six types of questions also reaches 80% or more of the total score of this aspect (17).

#### 2.3.2 Setting

The questionnaire was developed by the China Health Education Center commissioned by the National Health Commission of the People’s Republic of China and used to survey the health literacy level of residents in China. It has high authority and has been widely used. The Cronbach’s *α* coefficient is 0.82–0.931, and the split-half reliability is 0.808–0.81 (18, 19), which has good reliability and validity. The topic comes from the content of health literacy of Chinese citizens, and the health literacy of college students is investigated from different aspects, which is targeted and comprehensive. This study used the “Questionnaire Star” (online questionnaire survey platform) to conduct the survey, before distributing the questionnaire to the research subjects to explain the purpose and significance of the study, to obtain the consent of the research subjects after the distribution of the questionnaire, each person can only fill in the questionnaire once, and complete all questions before submission. In the data processing stage, unqualified questionnaires with short or long response time (according to ^−^x ± s, < 219.62 s or > 1062.51 s), regular responses or abnormal values were eliminated, and the data were checked by two investigators and entered.

#### 2.3.3 Data analyses

An electronic questionnaire survey was conducted and a database was formed. SPSS 22.0 software was used for data processing and analysis. Descriptive statistical analysis was used to analyze the general data of the respondents, chi-square test was used to compare the count data between groups, and Logistic regression model was used to analyze the influencing factors of health literacy. The test level *α* = 0.05.

## 3 Results

### 3.1 Participants’ characteristics

A total of 1,092 electronic questionnaires were collected in this survey, of which 925 were valid questionnaires, with an effective recovery rate of 84.7%. The Cronbach’s *α* coefficient of the questionnaire in this study was 0.873, and the Spearman-Brown coefficient was 0.799. The internal consistency of the questionnaire was good. Males accounted for 26.81% and females accounted for 73.19%. In terms of majors, the major was medicine, accounting for 70.70%; most of the students were not the only child (73.19%), and their close relatives were not engaged in the medical profession (83.24%). The parents’ educational background was generally low, high school or below accounted for 74.59 and 78.92%, respectively. 66.38% of college students had normal body mass index (BMI). Most of the college students never smoked (94.05%), and 78.05% of them were aware of the concept of “health literacy” (see Table 1 for details).

**Table 1:** General characteristics of participants (*n* = 925).

Characteristics		Number [cases (%)]	Characteristics		Number [cases (%)]
Gender	Male	248 (26.81)	Mother’s education background	High school and below	730 (78.92)
	Female	677 (73.19)		College degree	113 (12.22)
Nation	Han Chinese	906 (97.95)		Bachelor degree	72 (7.78)
	Ethnic minorities	19 (2.05)		Master’s degree or above	10 (1.08)
Grade	Freshman year	311 (33.62)	Monthly living expenses (yuan)	≤500	19 (2.05)
	Sophomore year	138 (14.92)		501~	79 (8.54)
	Junior year	430 (46.49)		1,001~	402 (43.46)
	Senior year	46 (4.97)		1,501~	341 (36.86)
Major	Non-medical	271 (29.30)		≥2001	84 (9.08)
	Medical	654 (70.70)	Family population	1 ~ 3	265 (28.65)
Home address	Cities	296 (32.00)		4 ~ 5	582 (62.92)
	Towns and cities	198 (21.41)		≥6	78 (8.43)
	Rural areas	431 (46.59)	BMI	Lean and thin	163 (17.62)
Only child	Yes	248 (26.81)		Normal	614 (66.38)
	No	677 (73.19)		Overweight	112 (12.11)
Close relatives were in the medical profession	Yes	155 (16.76)		Obesity	36 (3.89)
	No	770 (83.24)	Smoking	Ever or current smoker	55 (5.95)
Father’s education background	High school and below	690 (74.59)		Never	870 (94.05)
	College degree	117 (12.65)	Being aware of the concept of “health literacy”	Yes	722 (78.05)
	Bachelor degree	103 (11.14)		No	203 (21.95)
	Master’s degree or above	15 (1.62)			

### 3.2 Overall level of health literacy

**Figure 1:**
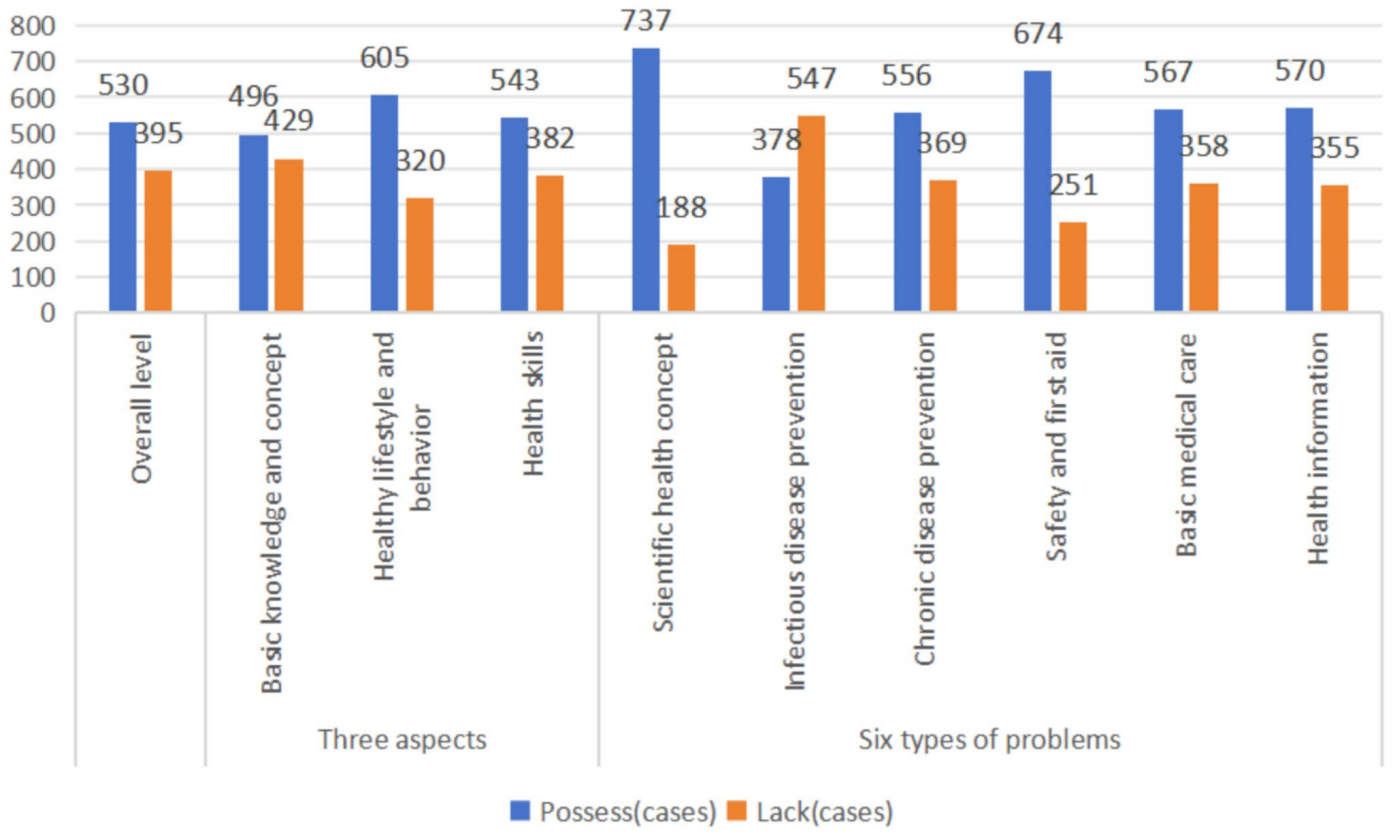
Overall level of health literacy, three aspects and six types of problems (*n* = 925).

The overall possession rate of health literacy was 57.30%. Among the three aspects of health literacy, the possession rates of college students’ basic knowledge and concept literacy, healthy lifestyle and behavior literacy, and basic skills literacy were 53.62, 65.41, and 58.70%, respectively, and the basic knowledge and concept literacy was the lowest. Among the six types of health literacy questions, college students had the highest literacy rate of scientific health concept, which was 79.68%. The awareness rate of infectious disease prevention and treatment was the lowest (40.86%). The awareness rate of chronic disease prevention and control literacy was 60.11%. The rate of safety and first aid literacy was 72.86%; the rate of basic medical literacy was 61.30%; the rate of health information literacy was 61.62% (see Figure 1 for details).

### 3.3 Single factor analysis of influencing factors of health literacy level

The analysis results showed that the overall level of health literacy of college students was significantly related to gender (*χ^2^* = 30.99, *p* < 0.001), grade (*χ^2^* = 17.85, *p* < 0.001), major (*χ^2^* = 5.65, *p* = 0.017), smoking (*χ^2^* = 24.23, *p* < 0.001) and being aware of the concept of “health literacy” (*χ^2^* = 7.73, *p* = 0.005) were statistically significant. The three aspects and six kinds of problems of health literacy of college students in the TCM university shows the following characteristics: females students are higher than males students, students of Han Chinese have higher literacy level, sophomore students have the lowest literacy level, medical students have higher literacy level, students with too much or too little living expenses have lower literacy level, students who never smoked have higher literacy level, and students who know the concept of “health literacy” have higher literacy level. The details are shown in Tables 2, 3.

**Table 2:** Single factor analysis of influencing factors of health literacy level [cases (%)].

Content of the survey	Health literacy level [cases (%)]	χ^2^-value	*p*-value	Content of the survey	Health literacy level [cases (%)]	χ^2^-value	*p*-value
Gender		30.99	<0.001	Mother’s education background		6.52	0.089
Male	105 (42.34)			High school and below	413 (56.58)		
Female	425 (62.78)			College degree	76 (67.26)		
Nation		1.83	0.176	Bachelor degree	36 (50.00)		
Han Chinese	522 (57.62)			Master’s degree or above	5 (50.00)		
Ethnic minorities	8 (42.11)			Monthly living expenses (yuan)		8.81	0.066
Grade		17.85	<0.001	≤500	6 (31.58)		
Freshman year	175 (56.27)			501~	49 (62.03)		
Sophomore year	59 (42.75)			1,001~	237 (58.96)		
Junior year	264 (61.40)			1,501~	197 (57.77)		
Senior year	32 (69.57)			≥2001	41 (48.81)		
Major		5.65	0.017	Family population		1.11	0.574
Non-medical	139 (51.29)			1 ~ 3	149 (56.23)		
Medical	391 (59.79)			4 ~ 5	332 (57.04)		
Home address		1.88	0.391	≥6	49 (62.82)		
Cities	160 (54.05)			BMI		3.48	0.324
Towns and cities	116 (58.59)			Lean and thin	95 (58.28)		
Rural areas	254 (58.93)			Normal	359 (58.47)		
Only child		1.48	0.224	Overweight	60 (53.57)		
Yes	134 (54.03)			Obesity	16 (44.44)		
No	396 (58.49)			Smoking		24.23	<0.001
Close relatives were in the medical profession		0.32	0.570	Ever or current smoker	14 (25.45)		
Yes	92 (59.35)			Never	516 (59.31)		
No	438 (56.88)			Being aware of the concept of “health literacy”		7.73	0.005
Father’s education background		1.56	0.668	Yes	431 (59.70)		
High school and below	391 (56.67)			No	99 (48.77)		
College degree	73 (62.39)						
Bachelor degree	57 (55.34)						
Master’s degree or above	9 (60.00)						

**Table 3:** Single factor analysis of influencing factors of three aspects and six types of problems levels of health literacy [cases (%)].

Content of the survey	Three aspects			Six types of problems					
	Basic knowledge and concept	Healthy lifestyle and behavior	Health skills	Scientific health concept	Infectious disease prevention	Chronic disease prevention	Safety and first aid	Basic medical care	Health information
Gender									
Male	113 (45.56)	125 (50.40)	110 (44.35)	171 (68.95)	77 (31.05)	123 (49.60)	159 (64.11)	119 (47.98)	123 (49.60)
Female	383 (56.57)	480 (70.90)	433 (63.96)	566 (83.60)	301 (44.46)	433 (63.96)	515 (76.07)	448 (66.17)	447 (66.03)
χ^2^-value	8.85	33.71	28.77	24.07	13.51	15.61	13.13	25.32	20.72
*p*-value	0.003	<0.001	<0.001	<0.001	<0.001	<0.001	<0.001	<0.001	<0.001
Nation									
Han Chinese	489 (53.97)	595 (65.67)	532 (58.72)	721 (79.58)	370 (40.84)	547 (60.38)	659 (72.74)	560 (61.81)	557 (61.48)
Ethnic minorities	7 (36.84)	10 (52.63)	11 (57.89)	16 (84.21)	8 (42.11)	9 (47.37)	15 (78.95)	7 (36.84)	13 (68.42)
χ^2^-value	2.20	1.40	0.01	0.04	0.01	1.31	0.36	4.89	0.38
*p*-value	0.138	0.237	0.942	0.835	0.912	0.252	0.547	0.027	0.538
Grade									
Freshman year	164 (52.73)	205 (65.92)	188 (60.45)	262 (84.24)	142 (45.66)	171 (54.98)	223 (71.70)	182 (58.52)	192 (61.74)
Sophomore year	57 (41.30)	74 (53.62)	68 (49.28)	102 (73.91)	53 (38.41)	75 (54.35)	91 (65.94)	72 (52.17)	63 (45.65)
Junior year	247 (57.44)	294 (68.37)	256 (59.53)	338 (78.60)	165 (38.37)	279 (64.88)	321 (74.65)	284 (66.05)	282 (65.58)
Senior year	28 (60.87)	32 (69.57)	31 (67.39)	35 (76.09)	18 (39.13)	31 (67.39)	39 (84.78)	29 (63.04)	33 (71.74)
χ^2^-value	12.01	10.53	7.01	7.51	4.47	10.42	7.56	10.00	19.73
*p*-value	0.007	0.015	0.072	0.057	0.215	0.015	0.056	0.019	<0.001
Major									
Non-medical	133 (49.08)	157 (57.93)	139 (51.29)	209 (77.12)	104 (38.38)	148 (54.61)	179 (66.05)	147 (54.24)	136 (50.18)
Medical	363 (55.50)	448 (68.50)	404 (61.77)	528 (80.73)	274 (41.90)	408 (62.39)	495 (75.69)	420 (64.22)	434 (66.36)
χ^2^-value	3.18	9.46	8.68	1.54	0.98	4.83	9.00	8.04	21.20
*p*-value	0.074	0.002	0.003	0.214	0.322	0.028	0.003	0.005	<0.001
Home address									
Cities	149 (50.34)	183 (61.82)	163 (55.07)	229 (77.36)	121 (40.88)	174 (58.78)	213 (71.96)	164 (55.41)	171 (57.77)
Towns and cities	116 (58.59)	132 (66.67)	119 (60.10)	163 (82.32)	86 (43.43)	125 (63.13)	144 (72.73)	122 (61.62)	131 (66.16)
Rural areas	231 (53.60)	290 (67.29)	261 (60.56)	345 (80.05)	171 (39.68)	257 (59.63)	317 (73.55)	281 (65.20)	268 (62.18)
χ^2^-value	3.25	2.49	2.38	1.87	0.79	1.01	0.23	7.10	3.64
*p*-value	0.197	0.288	0.304	0.393	0.673	0.603	0.893	0.029	0.162
Only child									
Yes	129 (52.02)	151 (60.89)	136 (54.84)	192 (77.42)	96 (38.71)	143 (57.66)	172 (69.35)	140 (56.45)	156 (62.90)
No	367 (54.21)	454 (67.06)	407 (60.12)	545 (80.50)	282 (41.65)	413 (61.00)	502 (74.15)	427 (63.07)	414 (61.15)
χ^2^-value	0.35	3.06	2.09	1.07	0.65	0.85	2.11	3.35	0.24
*p*-value	0.553	0.080	0.149	0.302	0.420	0.358	0.146	0.067	0.628
Close relatives were in the medical profession									
Yes	85 (54.84)	103 (66.45)	93 (60.00)	117 (75.48)	65 (41.94)	92 (59.35)	110 (70.97)	98 (63.23)	96 (61.94)
No	411 (53.38)	502 (65.19)	450 (58.44)	620 (80.52)	313 (40.65)	464 (60.26)	564 (73.25)	469 (60.91)	474 (61.56)
χ^2^-value	0.11	0.09	0.13	2.02	0.09	0.04	0.34	0.29	0.01
*p*-value	0.739	0.764	0.719	0.155	0.766	0.834	0.560	0.589	0.930
Father’s education background									
High school and below	364 (52.75)	454 (65.80)	410 (59.42)	548 (79.42)	283 (41.01)	414 (60.00)	504 (73.04)	428 (62.03)	423 (61.30)
College degree	67 (57.26)	76 (64.96)	71 (60.68)	94 (80.34)	51 (43.59)	73 (62.39)	89 (76.07)	71 (60.68)	80 (68.38)
Bachelor degree	56 (54.37)	64 (62.14)	54 (52.43)	82 (79.61)	37 (35.92)	58 (56.31)	69 (66.99)	59 (57.28)	60 (58.25)
Master’s degree or above	9 (60.00)	11 (73.33)	8 (53.33)	13 (86.67)	7 (46.67)	11 (73.33)	12 (80.00)	9 (60.00)	7 (46.67)
χ^2^-value	1.10	0.96	2.19	0.51	1.62	1.97	2.80	0.89	4.20
*p*-value	0.777	0.811	0.534	0.916	0.656	0.578	0.423	0.829	0.241
Mother’s education background									
High school and below	386 (52.88)	479 (65.62)	428 (58.63)	581 (79.59)	299 (40.96)	439 (60.14)	531 (72.74)	456 (62.47)	444 (60.82)
College degree	71 (62.83)	78 (69.03)	75 (66.37)	94 (83.19)	51 (45.13)	72 (63.72)	91 (80.53)	67 (59.29)	83 (73.45)
Bachelor degree	35 (48.61)	42 (58.33)	35 (48.61)	56 (77.78)	24 (33.33)	43 (59.72)	46 (63.89)	39 (54.17)	38 (52.78)
Master’s degree or above	4 (40.00)	6 (60.00)	5 (50.00)	6 (60.00)	4 (40.00)	2 (20.00)	6 (60.00)	5 (50.00)	5 (50.00)
χ^2^-value	5.49	2.39	6.08	3.41	2.55	7.33	7.14	2.69	9.84
*p*-value	0.139	0.496	0.108	0.332	0.467	0.062	0.068	0.441	0.020
Monthly living expenses (yuan)									
≤500	9 (47.37)	5 (26.32)	8 (42.11)	11 (57.89)	3 (15.79)	9 (47.37)	12 (63.16)	5 (26.32)	11 (57.89)
501~	51 (64.56)	52 (65.82)	54 (68.35)	63 (79.75)	32 (40.51)	46 (58.23)	60 (75.95)	48 (60.76)	53 (67.09)
1,001~	213 (52.99)	272 (67.66)	253 (62.94)	324 (80.60)	164 (40.80)	244 (60.70)	301 (74.88)	266 (66.17)	254 (63.18)
1,501~	185 (54.25)	228 (66.86)	186 (54.55)	274 (80.35)	147 (43.11)	211 (61.88)	250 (73.31)	202 (59.24)	209 (61.29)
≥2001	38 (45.24)	48 (57.14)	42 (50.00)	65 (77.38)	32 (38.10)	46 (54.76)	51 (60.71)	46 (54.76)	43 (51.19)
χ^2^-value	6.59	16.60	13.22	6.15	5.93	2.91	8.42	15.95	5.41
*p*-value	0.159	0.002	0.010	0.188	0.205	0.574	0.078	0.003	0.248
Family population									
1~3	142 (53.58)	165 (62.26)	155 (58.49)	208 (78.49)	109 (41.13)	159 (60.00)	186 (70.19)	152 (57.36)	166 (62.64)
4~5	317 (54.47)	384 (65.98)	337 (57.90)	456 (78.35)	233 (40.03)	344 (59.11)	429 (73.71)	364 (62.54)	355 (61.00)
≥6	37 (47.44)	56 (71.79)	51 (65.38)	73 (93.59)	36 (46.15)	53 (67.95)	59 (75.64)	51 (65.38)	49 (62.82)
χ^2^-value	1.37	2.65	1.60	10.19	1.08	2.25	1.48	2.66	0.26
*p*-value	0.505	0.266	0.451	0.006	0.584	0.326	0.478	0.264	0.878
BMI									
Lean and thin	81 (49.69)	102 (62.58)	99 (60.74)	129 (79.14)	64 (39.26)	97 (59.51)	117 (71.78)	94 (57.67)	98 (60.12)
Normal	348 (56.68)	407 (66.29)	364 (59.28)	485 (78.99)	255 (41.53)	378 (61.56)	455 (74.10)	392 (63.84)	386 (62.87)
Overweight	48 (42.86)	76 (67.86)	64 (57.14)	93 (83.04)	41 (36.61)	65 (58.04)	78 (69.64)	65 (58.04)	64 (57.14)
Obesity	19 (52.78)	20 (55.56)	16 (44.44)	30 (83.33)	18 (50.00)	16 (44.44)	24 (66.67)	16 (44.44)	22 (61.11)
χ^2^-value	8.55	2.63	3.50	1.29	2.37	4.45	1.86	7.40	1.51
*p*-value	0.036	0.453	0.321	0.733	0.499	0.217	0.602	0.060	0.680
Smoking									
Ever or current smoker	18 (32.73)	20 (36.36)	17 (30.91)	33 (60.00)	12 (21.82)	18 (32.73)	30 (54.55)	13 (23.64)	17 (30.91)
Never	478 (54.94)	585 (67.24)	526 (60.46)	704 (80.92)	366 (42.07)	538 (61.84)	644 (74.02)	554 (63.68)	553 (63.56)
χ^2^-value	10.27	21.80	18.63	13.98	8.78	18.28	9.93	34.96	23.32
*p*-value	0.001	<0.001	<0.001	<0.001	0.003	<0.001	0.002	<0.001	<0.001
Being aware of the concept of “health literacy”									
Yes	412 (57.06)	480 (66.48)	448 (62.05)	575 (79.64)	308 (42.66)	443 (61.36)	534 (73.96)	461 (63.85)	476 (65.93)
No	84 (41.38)	125 (61.58)	95 (46.80)	162 (79.80)	70 (34.48)	113 (55.67)	140 (68.97)	106 (52.22)	94 (46.31)
χ^2^-value	15.67	1.69	15.20	<0.01	4.38	2.14	2.00	9.04	25.80
*p*-value	<0.001	0.194	<0.001	0.959	0.036	0.143	0.157	0.003	<0.001

### 3.4 Logistic regression analysis was used to analyze the influencing factors of health literacy

Taking health literacy as the dependent variable and the influencing factors (gender, grade, major, smoking, and being aware of the concept of “health literacy”) with statistical significance in univariate analysis as the independent variables, Logistic regression analysis was conducted on the influencing factors of health literacy level. The results showed that females had a 92% more odds of higher health literacy than males (AOR: 1.92; 95%CI: 1.40–2.62). The health literacy level of sophomores was 0.62 times that of freshmen (AOR: 0.62; 95%CI: 0.41–0.95) and seniors had a 102% more odds of higher health literacy than freshmen (AOR: 2.02; 95%CI: 1.01–4.05). College students who never smoked had a 199% more odds of higher health literacy than among former or current smokers (AOR: 2.99; 95%CI: 1.57–5.72). College students who were aware of the concept of “health literacy” had a 54% more odds of higher health literacy than those who were not aware (AOR: 1.54; 95%CI: 1.11–2.13) (see Table 4).

**Table 4:** Logistic regression analysis of influencing factors of health literacy of college students.

Factors	Univariate analysis			Multivariate analysis		
	*p*-value	COR value	95%CI	*p*-value	AOR value	95%CI
Gender						
Male (reference)						
Female	<0.001	2.30	1.71 ~ 3.09	<0.001	1.92	1.40 ~ 2.62
Grade						
Freshman year (reference)						
Sophomore year	0.008	0.58	0.39 ~ 0.87	0.027	0.62	0.41 ~ 0.95
Junior year	0.161	1.24	0.92 ~ 1.66	0.251	1.20	0.88 ~ 1.63
Senior year	0.091	1.78	0.91 ~ 3.46	0.048	2.02	1.01 ~ 4.05
Major						
Non-medical (reference)						
Medical	0.018	1.41	1.06 ~ 1.88			
Smoking						
Yes (reference)						
No	<0.001	4.27	2.29 ~ 7.95	0.001	2.99	1.57 ~ 5.72
Being aware of the concept of “health literacy”						
No (reference)						
Yes	0.006	1.56	1.14 ~ 2.13	0.009	1.54	1.11 ~ 2.13

## 4 Discussion

### 4.1 The overall level of health literacy of students in universities of TCM is relatively high

This study shows that the overall level of health literacy of college students in this TCM university is 57.30%, which is significantly higher than the level of health literacy of Chinese residents in 2021 (25.40%) (20). It is also higher than the health literacy level of college students in a survey in the United States in 2020 (49%) (21), 20 universities in China in 2020 (41.7%) (22), and 5 universities in Shaanxi Province in China in 2022 (39.2%) (23). Compared with other medical colleges and universities, the overall level of health literacy of college students in this study is also relatively high (24, 25), but it is lower than the health literacy level of college students in a university in Denmark in 2020 (59.9%) (8). Analysis of previous studies found that women’s health literacy was generally higher than that of men (10, 21, 23), and the proportion of women in this study (73.19%) was higher, which may lead to the improvement of the overall level of health literacy. Generally speaking, the higher the economic level, the higher the attention to health (11). Social culture may also affect the level of health literacy, and studies have shown that women have lower health literacy in patriarchal societies (11). In addition, medical students generally have higher health literacy than non-medical students (10, 11).

### 4.2 To improve the health literacy level of students in TCM colleges and universities according to the influencing factors

From the analysis of three aspects of health literacy, the healthy lifestyle and behavior literacy level of college students of TCM was the highest (65.41%), suggesting that although the lifestyle of college students of TCM has changed due to the medical atmosphere in school or the impact of COVID-19 pandemic (10, 26), the basic knowledge and concepts and health skills have not been improved. It shows that college students of TCM need to consolidate their theory and skills training, and integrate knowledge and practice. The results showed that males students, sophomore students, non-medical major, high or low monthly living expenses, ever or current smoking, and unawareness of the concept of “health literacy” were the negative factors for the three aspects of health literacy. The healthy lifestyle and behavior literacy level of students with monthly living expenses ≤500 (26.32%) is even lower than that of national residents in 2021 (28.05%) (20). Personal economic level may affect their own health care access and use (11), suggesting that schools should focus on these students, form targeted intervention programs according to the “knowledge, attitude and practice” model, carry out health literacy related courses or practical activities, popularize medical knowledge and skills to non-medical students, increase efforts to publicize the disadvantages of smoking and strictly implement smoking cessation control. Schools or society should provide subsidies to students with life difficulties, and improve their health literacy level as soon as possible. According to the analysis of the six questions of health literacy, the literacy level of infectious disease prevention and treatment among college students of TCM was the lowest (40.86%), which was lower than that of students in a western medicine university (45.5%) (25). Perhaps due to the differences in curriculum and philosophy between Chinese and western medicine universities, the awareness of disinfection and isolation of students in TCM universities was weaker than that of students in western medicine universities. It may also be related to the lack of education related to the prevention and control of infectious diseases in schools, and students are less faced with the prevention and control of infectious diseases and lack of practical combat experience (25), suggesting that this aspect is the deficiency of college students in TCM colleges and universities. Schools should strengthen the education of infectious disease prevention and control among students and carry out exercises to improve their health literacy level.

Logistic regression analysis showed that gender, grade, smoking, and being aware of the concept of “health literacy” were significantly associated factors affecting the level of health literacy of TCM college students. The health literacy level of females is almost twice that of males, which is consistent with the higher health literacy level of females in the 2022 survey results of the health literacy level of students in 5 colleges and universities in Shaanxi Province of China (23) which may be related to females’ more active attention to health information. However, the results of the health literacy survey of undergraduates in a university in Nepal in 2021 showed that females were 1.6 times more likely to have poor health literacy than males (11), which may be related to the regional economic level, social culture, school differences, professional curriculum Settings, etc. The health literacy level of non-smoking undergraduates is almost three times higher than that of former or current smokers. A cross-sectional survey of health literacy of students in a public university in northern Jordan also showed that non-smoking students have higher health literacy level (10), which may be related to non-smoking students’ strong self-restraint and more emphasis on their own health (27). Therefore, it is necessary to implement more effective tobacco control measures in schools. A survey on the intention and behavior of tobacco control among college students in 12 universities in China found that improving the performance expectation, effort expectation and eHealth literacy of non-smoking college students and creating a positive social environment can improve the tobacco control behavior of college students (27), and the measures taken by this school provide a reference. The health literacy level of senior students is higher than that of freshman students, which is consistent with the research results of undergraduate health literacy of 10 universities in Tianjin, China in 2021 (28), mainly due to the fact that senior students have more knowledge reserve, social ability and personal experience. However, the results of this survey show that the health literacy level of sophomore students is slightly lower than that of freshman students, which is not consistent with the research expectation. The analysis may be related to the sample size, and may also be due to the fact that freshman students have just entered the university and still maintain a strong sense of self-discipline and health awareness (29). Students who are aware of the concept of “health literacy” have a higher level of health literacy, which may be due to their strong willingness and ability to obtain health information, and they can actively collect health-related information from friends, newspapers, and the Internet (29). This indicates that schools need to set up health resources reasonably, carry out health education related activities and courses in a diversified way (30), form a new interdisciplinary talent training paradigm, and improve the health literacy level of college students.

To the best of our knowledge, this is the first study to explore the health literacy level and influencing factors of students in TCM colleges and universities in China, which provides a reference for researchers to explore the reasonable setting of health resources, carry out health education related activities and courses, and improve the health literacy level of students in TCM colleges and universities.

### 4.3 Limitations

The limitations of this study mainly include: firstly, this study was conducted in only one TCM college and the sample was not very representative. Second, due to the limitation of time and cost, the sampling method is a convenience sampling method, which may have errors. Although in order to reduce the bias caused by convenient sampling, researchers selected research subjects from different grades and classes as much as possible, collected demographic characteristics data, and analyzed the composition of the sample. Therefore, future researchers should conduct multi-center research with stratified random sampling method, and carry out effective intervention strategies to improve the health literacy level of college students.

## 5 Conclusion

Compared with previous studies, the health literacy level of 925 students in TCM colleges was above the middle level. The healthy lifestyle and behavior literacy of students in TCM colleges is better than knowledge and skill literacy, which is a separation phenomenon of knowledge and practice. Gender, grade, smoking status and awareness of the concept of “health literacy” were important factors affecting the level of health literacy. The results of this study and the analysis of influencing factors can provide reference for TCM colleges and universities to improve health education activities or curriculum, help to improve the health literacy level of students in TCM colleges and universities, give full play to the professional characteristics of TCM colleges and universities, and promote the construction of healthy schools.

## Data Availability

The raw data supporting the conclusions of this article will be made available by the authors, without undue reservation.

## References

[1] Bandura A Caprara GV Barbaranelli C Gerbino M Pastorelli C. Role of affective self-regulatory efficacy in diverse spheres of psychosocial functioning. Child Dev. (2003) 74:769–82. doi: 10.1111/1467-8624.00567, PMID: 12795389

[2] Grové C Marinucci A Riebschleger J. Development of an American and Australian co-designed youth mental health literacy program. Front Child Adolesc Psychiatry. (2023) 1:1018173. doi: 10.3389/frcha.2022.1018173, PMID: 39817280 PMC11731624

[3] Seangpraw K Ong-Artborirak P Boonyathee S Bootsikeaw S Kantow S Panta P. Effect of health literacy intervention on glycemic control and renal function among Thai older adults at risk of type 2 diabetes mellitus. Clin Interv Aging. (2023) 18:1465–76. doi: 10.2147/CIA.S413456, PMID: 37700781 PMC10494859

[4] Wang Y Xu X Zhang F. Construction of a theoretical framework of maternal mental health literacy based on the grounded theory. J Nurs Sci. (2024) 39:82–6. doi: 10.3870/j.issn.1001-4152.2024.15.082

[5] Lee EH Lee YW Lee KW Nam M Kim SH. A new comprehensive diabetes health literacy scale: development and psychometric evaluation. Int J Nurs Stud. (2018) 88:1–8. doi: 10.1016/j.ijnurstu.2018.08.002, PMID: 30142483

[6] The State Council of the People’s Republic of China. Circular of the general office of the state council on the issuance of the 14th five-year national health plan. (2022). Available at: https://www.gov.cn/zhengce/zhengceku/2022-05/20/content_5691424.htm (Accessed April 25, 2023).

[7] Ministry of Education of the People’s Republic of China. Circular of the general office of the ministry of education on the implementation of the national healthy school construction plan. (2022). Available at: http://www.moe.gov.cn/srcsite/A17/s7059/202204/t20220424_621280.html (Accessed May 22, 2023).

[8] Bak CK Krammer J Dadaczynski K Orkan O von Seelen J Prinds C. Digital health literacy and information-seeking behavior among university college students during the COVID-19 pandemic: a cross-sectional study from Denmark. Int J Environ Res Public Health. (2022) 19:3676. doi: 10.3390/ijerph19063676, PMID: 35329363 PMC8955471

[9] Sons A Eckhardt AL. Health literacy and knowledge of female reproduction in undergraduate students. J Am Coll Heal. (2023) 71:836–43. doi: 10.1080/07448481.2021.1909034, PMID: 33891527

[10] Rababah JA Al-Hammouri MM Drew BL Aldalaykeh M. Health literacy: exploring disparities among college students. BMC Public Health. (2019) 19:1401. doi: 10.1186/s12889-019-7781-2, PMID: 31664973 PMC6819582

[11] Bhusal S Paudel R Gaihre M Paudel K Adhikari TB Pradhan PMS. Health literacy and associated factors among undergraduates: a university-based cross-sectional study in Nepal. PLoS Glob Public Health. (2021) 1:e0000016. doi: 10.1371/journal.pgph.0000016, PMID: 36962072 PMC10022320

[12] Pourfridoni M Khan MA Daneshi S Vazirinasab H Nosrati Z Daneshi-Maskooni M. Health literacy and fear among Iranian medical students due to COVID-19: an observational study. Brain Behav. (2022) 12:e2586. doi: 10.1002/brb3.2586, PMID: 35429408 PMC9110898

[13] Stone M Bazaldua O Morrow J. Developing health literacy communication practices for medical students. MedEdPORTAL. (2021) 17:11091. doi: 10.15766/mep_2374-8265.11091, PMID: 33537408 PMC7842086

[14] Xu J Miao Y Fan Y Liu J. Study on the current situation and influencing factors of E-health literacy among collegians in traditional Chinese medicine universities. Health Vocat Educ. (2023) 41:121–4. doi: 10.20037/j.issn.1671-1246.2023.05.36

[15] Wu DY Hou XT Xia ZS Hao EW Xie JL Liang JY. Analysis on oral medication rules of traditional Chinese medicine prescriptions for prevention of COVID-19. Chin Herb Med. (2021) 13:502–17. doi: 10.1016/j.chmed.2021.10.007, PMID: 34659385 PMC8505017

[16] National Health Commission of the People’s Republic of China. Notice of the general office of the National Health and Family Planning Commission on the issuance of the health literacy of Chinese citizens-basic knowledge and skills (2015 Edition). (2015) Available at: http://www.nhc.gov.cn/xcs/s3581/201601/e02729e6565a47fea0487a212612705b.shtml (Accessed April 25, 2023).

[17] Nie X Li Y Li L. Statistic analysis of 2012 Chinese residents health literacy monitoring. Chin J Health Educ. (2014) 30:178–81. doi: 10.16168/j.cnki.issn.1002-9982.2014.02.021

[18] Du X Han T Jing C Zhuang R. Construction and verification of the National Residents' health literacy surveillance rapid assessment questionnaire (HLSRAQ). Health Educ Health Promot. (2019) 14:310–3. doi: 10.16117/j.cnki.31-1974/r.201904007

[19] Li Y Mao Q Shi Q Tao M Nie X Li L. The level of health literacy of Chinese residents in 2012: surveillance results. Chin J Health Educ. (2015) 31:99–103. doi: 10.16168/j.cnki.issn.1002-9982.2015.02.001

[20] National Health Commission of the People’s Republic of China. In 2021, the health literacy level of Chinese residents reached 25.40%. (2022). Available at: http://www.nhc.gov.cn/xcs/s3582/202206/5dc1de46b9a04e52951b21690d74cdb9.shtml (Accessed May 15, 2023).

[21] Patil U Kostareva U Hadley M Manganello JA Okan O Dadaczynski K. Health literacy, digital health literacy, and COVID-19 pandemic attitudes and behaviors in U.S. college students: implications for interventions. Int J Environ Res Public Health. (2021) 18:3301. doi: 10.3390/ijerph18063301, PMID: 33806763 PMC8004744

[22] Li S Cui G Kaminga AC Cheng S Xu H. Associations between health literacy, eHealth literacy, and COVID-19-related health behaviors among Chinese college students: cross-sectional online study. J Med Internet Res. (2021) 23:e25600. doi: 10.2196/25600, PMID: 33822734 PMC8104003

[23] Wu S Shao B Wang G. Health literacy among university students in Shaanxi Province of China: a cross-sectional study. Risk Manag Healthc Policy. (2023) 16:865–78. doi: 10.2147/RMHP.S407113, PMID: 37205003 PMC10185481

[24] Vamos S Lee T Kang HB Vamos CA. COVID-19 and college students: health literacy experiences and training needs. J Am Coll Heal. (2023) 71:2462–9. doi: 10.1080/07448481.2021.1970568, PMID: 34586044

[25] Wu Y Cao W Xu S. Study on the correlation between health literacy and health behavior: taking a medical College in Shanghai as an example. Health Vocat Educ. (2024) 42:63–7. doi: 10.20037/j.issn.1671-1246.2024.08.19

[26] Tang K Liang H. Lifestyle choices in the post-epidemic era. J Harbin Inst Technol. (2021) 23:50–7. doi: 10.16822/j.cnki.hitskb.2021.01.008

[27] Ma Y Zhou M Yu W Zou Z Ge P Ma ZF. Using the unified theory of acceptance and use of technology (UTAUT) and e-health literacy (e-HL) to investigate the tobacco control intentions and behaviors of non-smoking college students in China: a cross-sectional investigation. BMC Public Health. (2023) 23:765. doi: 10.1186/s12889-023-15644-5, PMID: 37098499 PMC10127360

[28] Li X. Health literacy and its influencing factors among college students in Tianjin in 2021. Chin J Prevent Control Chronic Dis. (2023) 31:66–70. doi: 10.16386/j.cjpccd.issn.1004-6194.2023.01.015

[29] Wang Y Hu Q Liu R Zhang H Chen J. Analysis on the status and influencing factors of health literacy of college students in Chengdu and Chongqing medical colleges. J Chengdu Med Col. (2022) 17:371–6. doi: 10.3969/j.issn.1674-2257.2022.03.021

[30] Yang HF Chang CC Tseng PL Lai HR Tasi JS Huang WH. Effectiveness of innovative instructional module for professional competence in health literacy in medical students. BMC Med Educ. (2022) 22:210. doi: 10.1186/s12909-022-03252-7, PMID: 35351115 PMC8960696

